# Foods and Dietaries

**Published:** 1891-03

**Authors:** 


*Foods and Dietaries.* By R. W. Burnet, M.D. London: Charles Griffin and Company. 1890.

We are not in love with diet tables, and this book is full of them: nevertheless, the author appears to have accomplished the difficult task of arranging a special diet for a very large number of distinct classes of disease; repetition is of course unavoidable, but patients who carry out the programme will not be likely to die of starvation unless they weary of the sameness of the diet from day to day. We think that the subject of feeding by enema might receive a more detailed consideration.

